# Long-term survival was achieved through multidisciplinary treatment of a patient with gallbladder carcinosarcoma accompanied by KRAS mutation: a case report and literature review

**DOI:** 10.3389/fonc.2024.1506949

**Published:** 2025-03-27

**Authors:** Yedan Liao, Junbo Lai, Jilan Yang, Tingfeng Long, Xi Wu, Dan Luo, Jin Tan, Ke Zhang, Jiadai Tang, Lin Xie

**Affiliations:** ^1^ Department of Gastroenterology Oncology, Yunnan Cancer Hospital, The Third Affiliated Hospital of Kunming Medical University, Peking University Cancer Hospital Yunnan, Kunming, China; ^2^ The Third Affiliated Hospital of Kunming Medical University & Clinical Oncology College, Kunming, China; ^3^ Pathology Department, Yunnan Cancer Hospital, The Third Affiliated Hospital of Kunming Medical University, Peking University Cancer Hospital Yunnan, Kunming, China; ^4^ Radiology Department, Yunnan Cancer Hospital, The Third Affiliated Hospital of Kunming Medical University, Peking University Cancer Hospital Yunnan, Kunming, China

**Keywords:** case report, carcinosarcoma of the gallbladder, KRAS mutation, diagnostic, treatment, prognosis

## Abstract

Carcinosarcoma of the gallbladder (CSGB) is an extremely rare subtype of primary gallbladder malignancy, which with a high rate of preoperative misdiagnosis due to its nonspecific clinical symptoms and imaging findings, as well as a high rate of recurrence within a short period of time after surgery, resulting in a very poor prognosis. Through a search of the medical literature, we found that there are few reports of CSGB receiving comprehensive treatments and achieving relatively good outcomes. Here, we report a rare case of CSGB with KRAS G12V mutation that achieved survival time of 32 months after receiving a combination of treatments including surgery, chemotherapy, radiotherapy, and immunotherapy, and we conducted a literature review for the disease with the aim of raising awareness of the disease.

## Introduction

CSGB, which first proposed by Landsteiner in 1907, is an rare subtype of primary malignant tumors of the gallbladder, accounting for less than 1% of gallbladder tumors, with a high degree of aggressiveness and poor prognosis (1, 2). A research of CSGB by Zhang et al. showed that the median age of onset of the disease was 68 years, the male to female ratio was approximately 1:3.25, the mean survival time was only 17.5 months, with a median survival of 5 months, and the 1-year and 5-year survival rates were (19 ± 5)% and (16 ± 5)%, respectively, patients with tumors <5 cm and Japanese nationality had longer survival times, the presence of gallstones, the type of epithelial and mesenchymal components, and patient’s age and gender are not important prognostic factors (2). The pathogenesis of carcinosarcoma is still unclear, but several theories have been proposed, including mesenchymal response, collision tumor hypothesis, malignant proliferation of epithelial origin, originating from embryonic quiescent cells, and full-potential stem cell hypothesis (3–6). Risk factors for the development of CSGB may be related to gallstones, chronic cholecystitis, and pancreaticobiliary malformation (PBM) (2, 7–9). Carcinosarcoma of the gallbladder has a high rate of underdiagnosis and misdiagnosis, as well as an extremely high rate of postoperative recurrence, making a poor prognosis of this disease in general. We report a case of a rare gallbladder carcinosarcoma with KRAS G12V mutation that underwent comprehensive treatment and achieved relatively long survival, and provide a literature review on this disease. The reporting of this case conforms to the CARE guidelines.

## Case presentation

### The initial symptoms of the patient

A 51-year-old female patient was admitted to the local hospital with epigastric pain.

The patient had been experiencing upper abdominal pain for over a month without radiating pain, nausea, or vomiting. No lumps were touched in the abdomen, and the patient felt no abdominal tenderness or rebound tenderness.

### Examination and treatment in external hospital

Computed tomography (CT) and Magnetic resonance imaging (MRI) examinations were performed and the results showed that gallbladder cancer was possible and the lesion invaded the bile ducts, and liver metastasis was possible. After receiving treatments for abdominal pain symptom, this patient came to our hospital for further clarification of disease diagnosis and access to treatment.

### Personal and family history

The patient had no hypertension, diabetes or cholecystitis in the past, and had no family history of oncological disease. She stated that her psychological condition was good.

### Laboratory and imaging examinations conducted in our hospital

**Figure 1:**
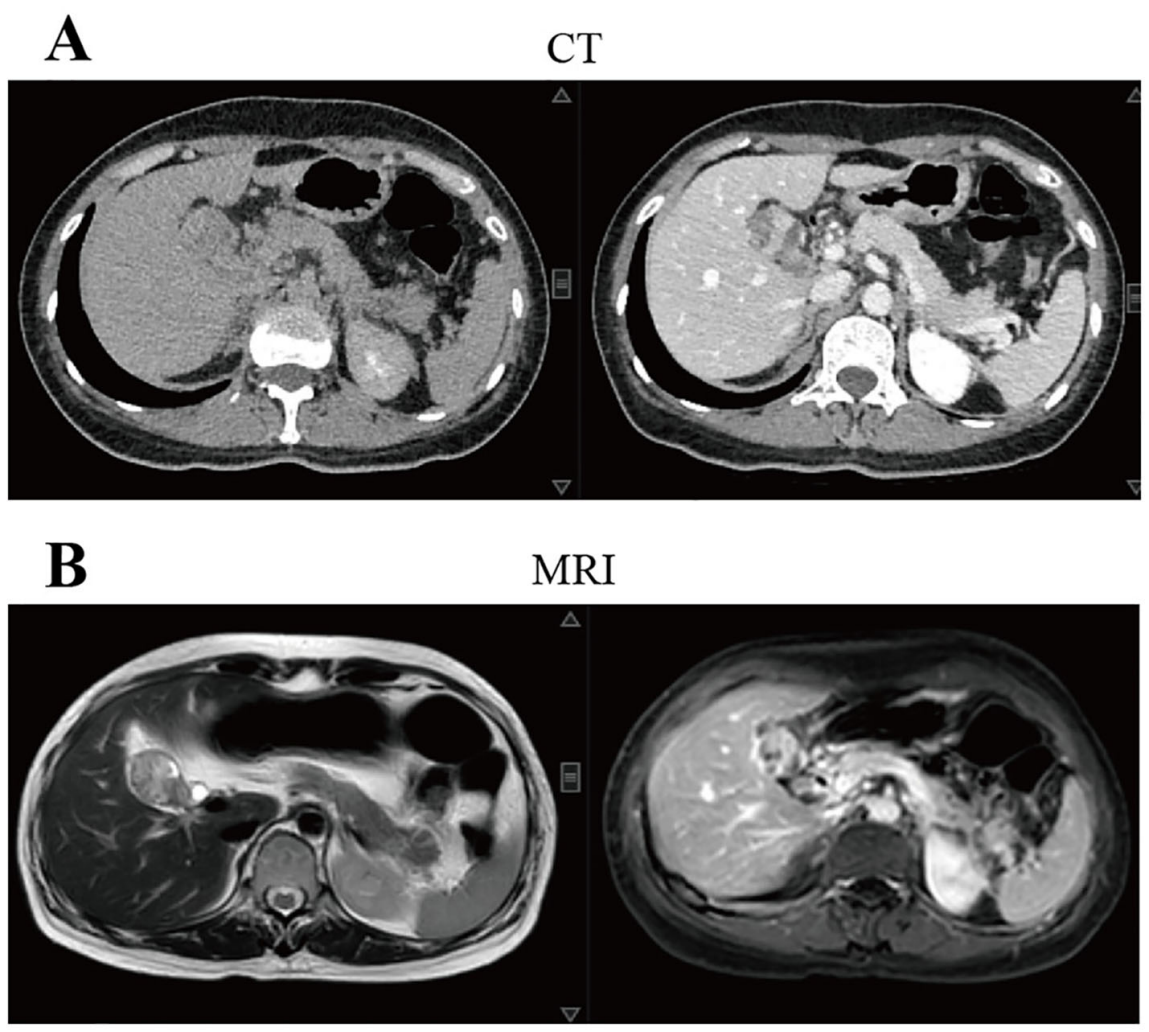
Imaging tests at the time of the patient’s first visit to our hospital suggested that gallbladder cancer was possible. **(A)** CT showed that the wall of the gallbladder was thickened and a mass measuring 3.6 cm×2.5 cm at the largest level was formed, with uneven enhancement after enhancement. The walls of the right and left intrahepatic bile ducts were slightly thicker and the lumen is slightly dilated; **(B)** MRI showed thickening of the wall of the gallbladder and formation of a mass with a maximum dimension of about 3.4 cm×2.2 cm, which was considered malignant in nature and was adjacent to the liver. The intrahepatic bile ducts and common hepatic duct were slightly dilated and the wall was slightly thick. CT, computed tomography; MRI, magnetic resonance imaging.

**Figure 2:**
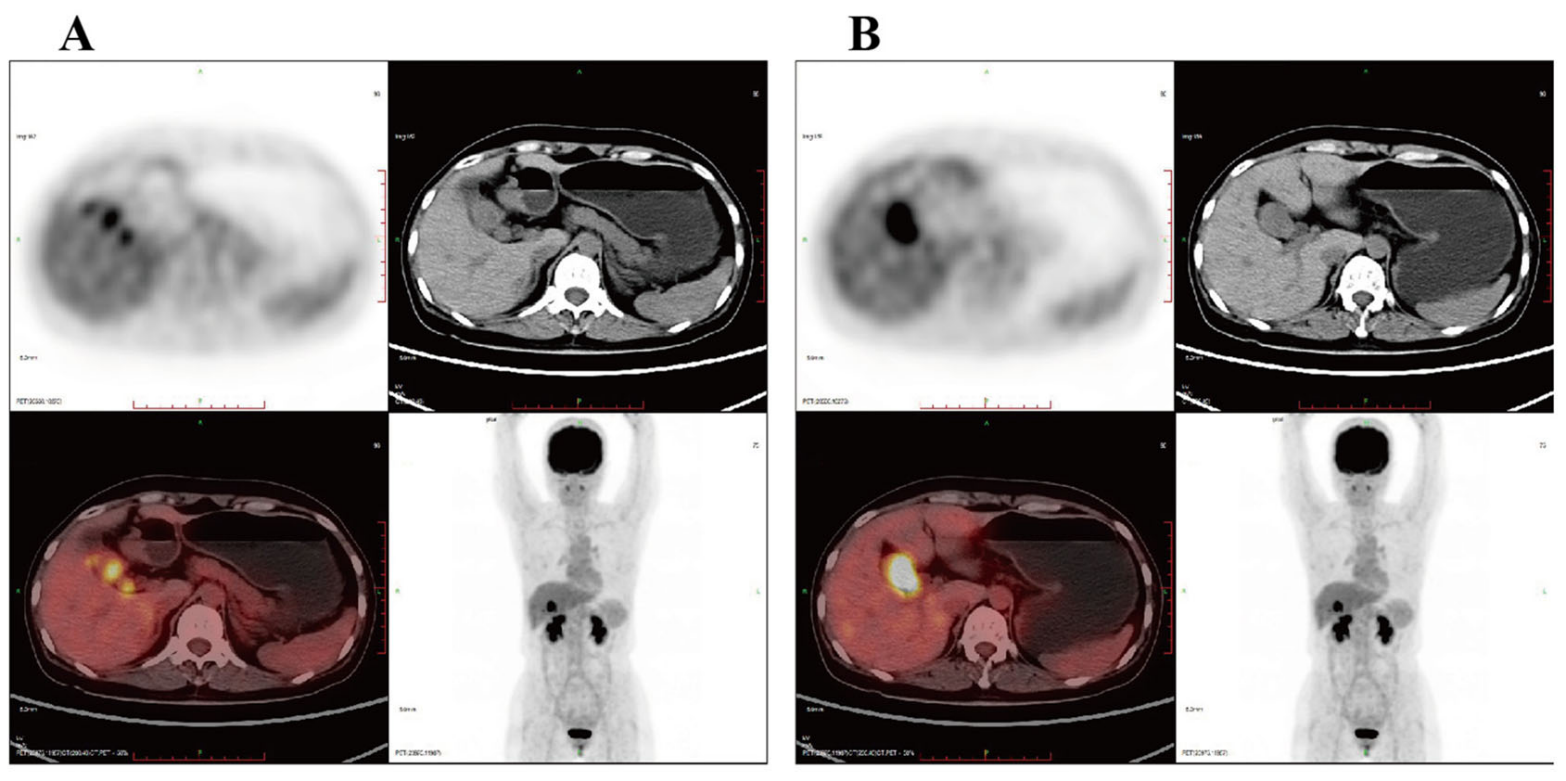
PET/CT suggested that the wall of the gallbladder was thickened to form a soft tissue mass with increased metabolism, which tended to malignant lesions with a high likelihood of gallbladder cancer. **(A)** The lesion involved the cystic duct and the upper part of the common bile duct; **(B)** mild dilatation of the intra- and extrahepatic bile ducts. PET/CT, positron emission tomography/computed tomography.

Both CT and MRI results (**Figure 1**) showed increased thickness of the gallbladder wall with a visible mass, and the possibility of gallbladder cancer was considered. Positron emission tomography/computed tomography (PET/CT) (**Figure 2**) suggested that the wall of the gallbladder was thickened to form a soft-tissue mass measuring about 3.8 cm×2.2 cm with increased FDG metabolism, which tended to be malignant, and gallbladder cancer was possible. Lesions involving the cystic duct and the upper part of the common bile duct, with mild dilatation of the intrahepatic and extrahepatic bile ducts.

### Surgery and postoperative adjuvant therapy

**Figure 3:**
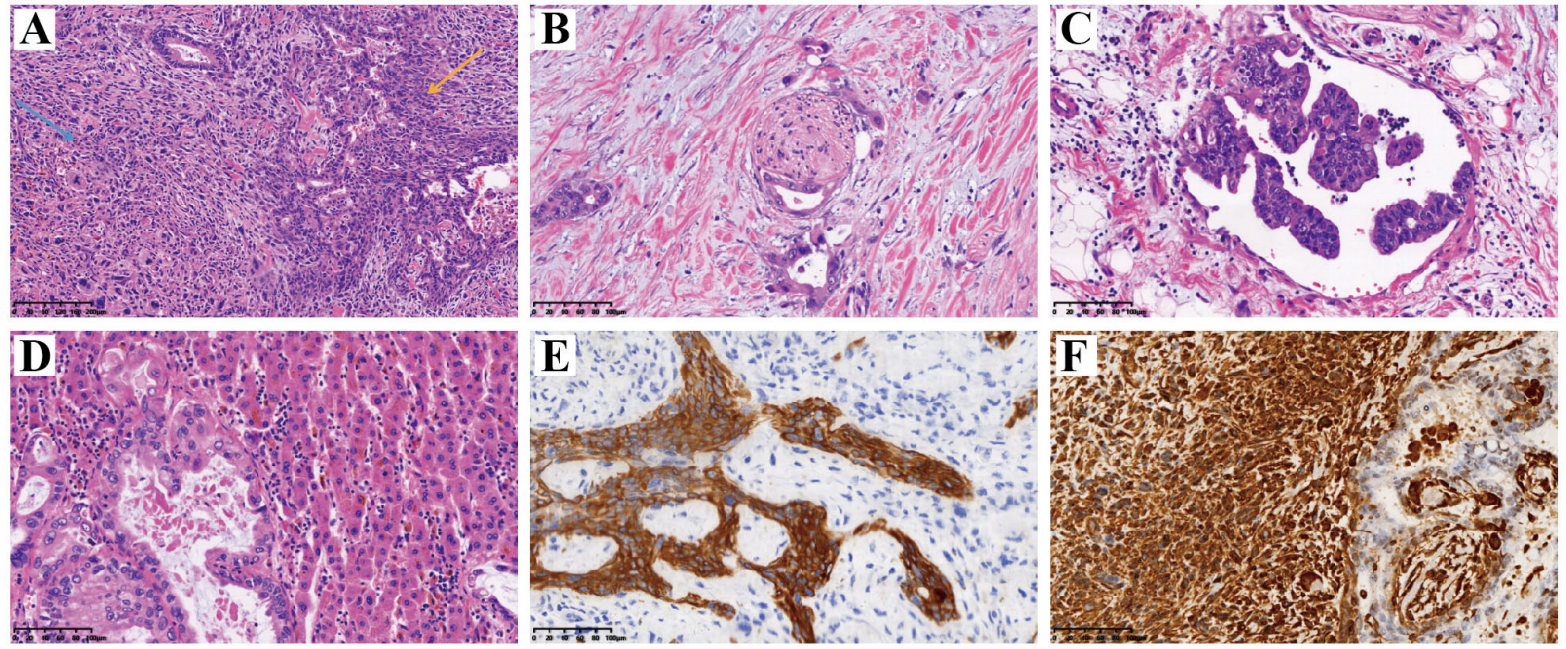
HE staining and immunohistochemical IHC staining of postoperative pathological tissues. **(A)** HE staining. Blue arrow indicated areas of adenocarcinoma and yellow arrow indicated areas of sarcoma. The adenoidal structure area and the spindle cell area were interspersed without clear demarcation, the cells were enlarged with obvious heterogeneity, the chromatin was rough, and a large number of nuclear divisions and multinucleated tumor giant cells could be seen; **(B)** Tumor cells infiltrated nerve fibers; **(C)** A vascular cancerous thrombus has formed; **(D)** The tumor has invaded the adjacent liver parenchyma; **(E)** Positive expression of CK in the epithelial component by IHC staining, which confirming its originate from epithelial tissue; **(F)** Positive expression of Vim in the sarcoma component by IHC staining, which confirming its originate from mesenchymal tissue. HE, hematoxylin-eosin; IHC, immunohistochemical; CK, cytokeratin; Vim, vimentin.

After the discussion by the hepatobiliary and pancreatic tumor MDT, “enlarged radical treatment of gallbladder cancer + resection of hepatoportal lesion + left and right hepatic duct plasty + Roux-en-Y hepatico-jejunostomy” was performed under general anesthesia. The postoperative cholecystectomy specimen was about 9 cm×5 cm×4 cm in size, grayish-red in color, and slightly soft in texture. HE staining suggested a malignant tumor of the gallbladder. The following indicators were examined using immunohistochemistry: CK (+), Vim (+), CK7 (+), CK20 (+), Villin (+), Syn (-), CgA (-), CD56 (-), CK19 (+), CD10 (+), AFP (-), Hepar1 (-), ki67 (+, 40%), SMA (-), desmin (-), CD34 (-), HMB45 (-), S100 (-), GFAP (-), Calponin (-), Myogenin (-), MyoD1 (-), CDX2 (-). The patient was diagnosed with carcinosarcoma which staging as phase IIIA when combined with HE and immunohistochemical findings, and microscopically showed intraluminal embolus in the vasculature and invasion of the nerve by the malignant tumor tissue. Representative meaningful HE and immunohistochemical pictures are shown in **Figure 3**.

After surgery, she received 6 cycles of “paclitaxel + isocyclophosphamide” chemotherapy and radiation therapy (PTV 46.8Gy/26F) targeting the postoperative tumor bed and lymph node drainage area. She had symptoms of IV degree myelosuppression with fever, small intestinal obstruction, and hepatic dysfunction, which were improved by symptomatic treatment, and his condition was stable after regular follow-up.

### Treatment of disease recurrence and complications

**Figure 4:**
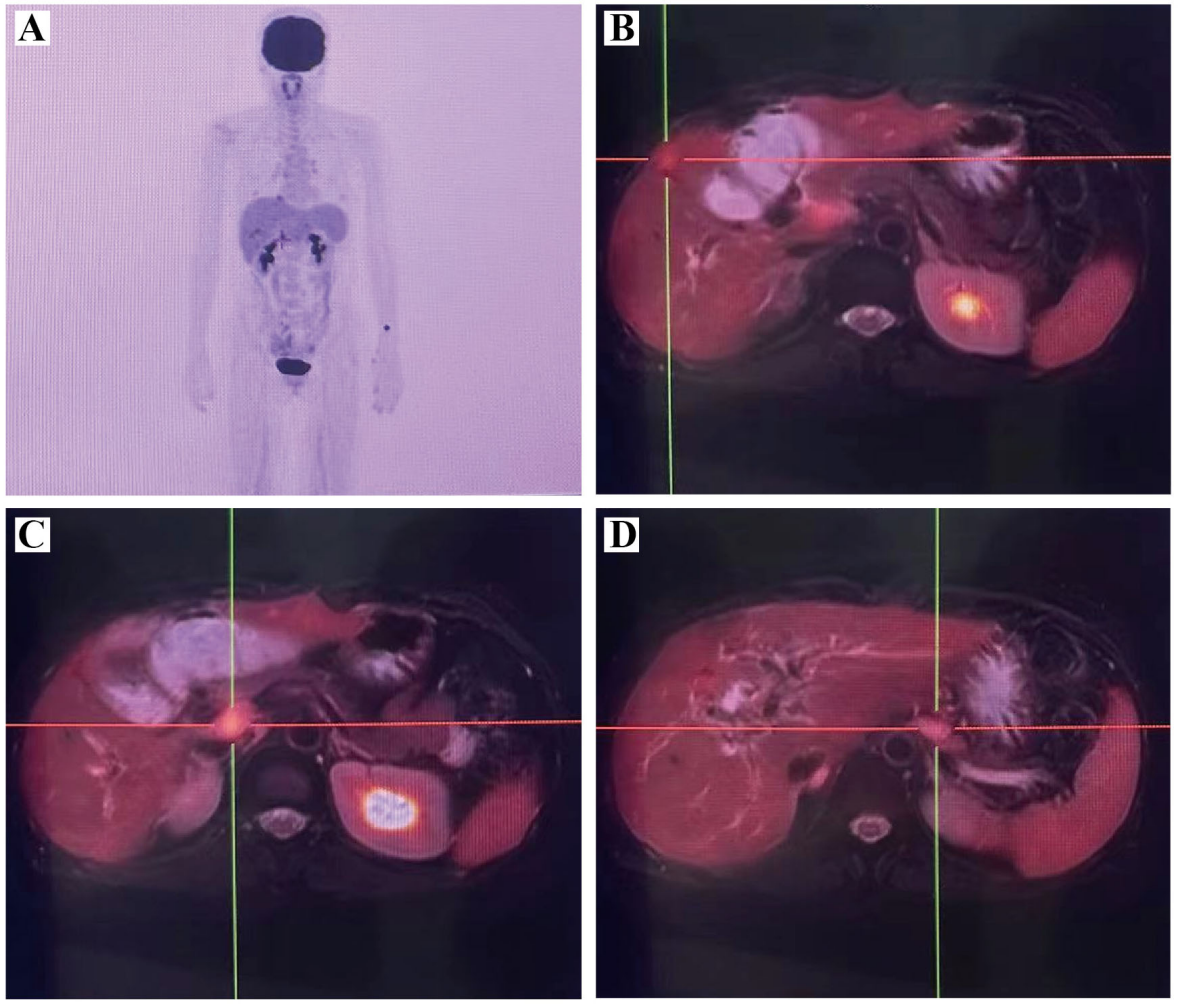
PET/MRI suggested recurrence and metastasis of gallbladder carcinosarcoma. **(A)** PET MIP image; **(B)** Multiple metastases in liver; **(C)** Lymph node metastasis in the hepatoportal area; **(D)** Lymph node metastasis in the gastric omental area. PET/MRI, positron emission tomography/magnetic resonance imaging.

**Figure 5:**
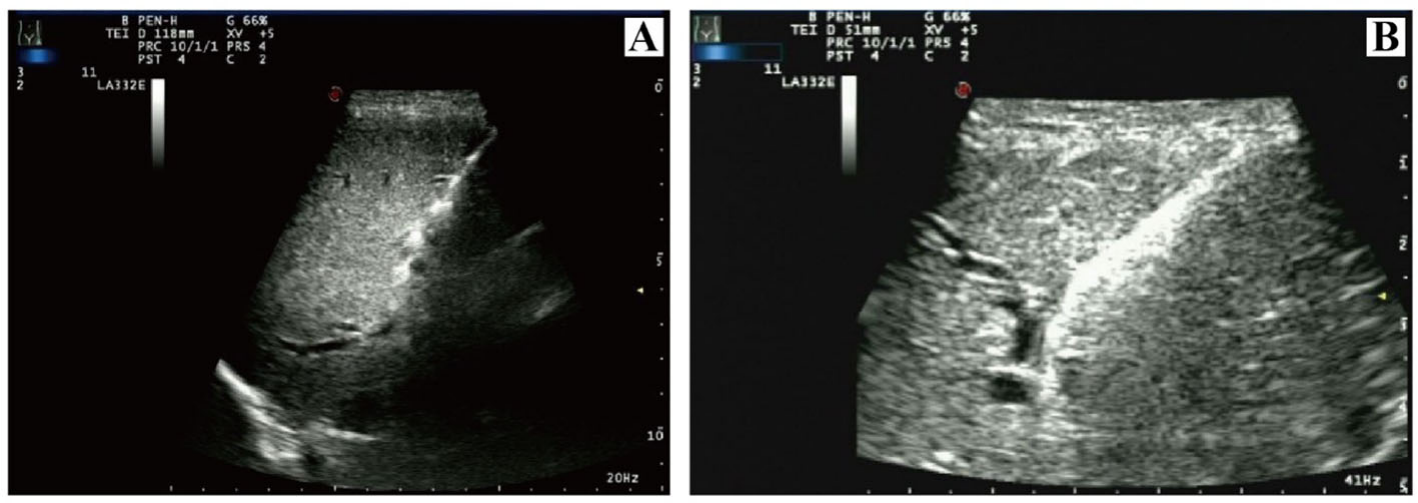
Ultrasound imaging of PTCD performed to improve jaundice and biliary tract infection. **(A)** initial catheter placement; **(B)** catheter replacement. PTCD, percutaneous transhepatic cholangial drainage.

Tumor markers were tested 14 months after surgery, and the test value of glycan antigen 19-9 was found to be 215.40 KU/L, which was significantly higher than the previous value. PET/MRI suggested postoperative changes in the bile ducts, elevated FDG in the marginal nodes of the operative area to consider tumor recurrence and intrahepatic metastasis, and multiple lymph node enlargement in the hilar and gastroretinal areas to consider metastasis (**Figure 4**). After consultation and discussion with experts, the patient presented with recurrence and metastasis after surgery for gallbladder cancer sarcoma, and was evaluated as having progressive disease. Genetic testing suggested that KRAS G12V, APC C4918T, TSC1 C1960G, TP53 C742T, BRCA1 were mutations, and there were no target drugs available. Six cycles of chemotherapy with the GP(Gemcitabine 1.2g d1,8; Cisplatinum 28mg d1-3, q21d) regimen were performed, plus immunotherapy with durvalumab. During this period, the patient had recurrent high fever with chills and high bilirubin values, and was considered to have a biliary tract infection, which was slightly improved by percutaneous transhepatic cholangial drainage (PTCD) (**Figure 5**) and anti-infective treatment. The patient was then given a change in chemotherapy to the XELOX regimen due to IV° myelosuppression, with intermittent oral targeted therapy with anlotinib during the same period. CT and MRI were regularly reviewed during this period and assessed tumor lesion as SD according to RECIST Version 1.1. Unfortunately, the patient passed away 32 months after the onset of the disease due to upper gastrointestinal bleeding with malignancy, severe biliary obstruction and infection.

### Patient perspective

**Figure 6:**
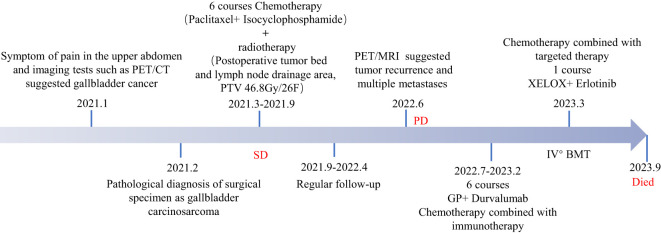
The timeline summarizing the main events of this case report.

**Figure 7:**
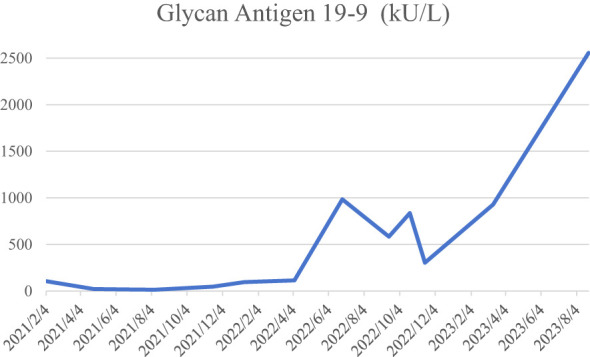
The trend of glycan antigen 19-9 during the course of the disease.

Like most patients with malignant tumors, she initially struggled to accept that she had such a rare disease. After accepting the fact, she agreed to have the operation and actively cooperated with the doctor for postoperative treatment. But there was no denying that her anxiety had increased significantly. So the doctor communicated with her more, told her to vent her emotions to reduce psychological pressure, and suggested that her family accompany her more. However, postoperative recurrence brought great shock to the patient. Bone marrow suppression and biliary obstruction also leaded to a decrease in the quality of life of patients. Throughout the entire process of disease diagnosis and treatment, the patient and her family members aware of the treatment plan and cooperated with the medical staff. A timeline of the major events reported in this case is shown in **Figure 6**. The trend of glycan antigen 19-9 during the course of the disease is shown in **Figure 7**.

## Discussion

Early symptoms of CSGB are not significant, Thomas et al. analyzed 78 cases of CSGB with abdominal pain (76.3%), Nonspecific symptoms such as weight loss, anorexia or lethargy (28.9%), nausea and vomiting (25.0%), fever in 13 cases (17.1%), and asymptomatic at the time of diagnosis in 2 cases (2.6%) (7). Similar to most patients, the initial symptom of the patient we reported was upper abdominal pain. Laboratory serologic tests for CSGB are nonspecific and more commonly include abnormal liver function. Tumor markers such as CA199, AFP, and CEA may be elevated or normal to assist in the diagnosis of CSGB, but have poor specificity. CT and MRI are more sensitive than ultrasound in detecting intrahepatic dissemination of malignant tumors, local lymph node involvement, and distant metastases, but CT does not distinguish well between gallbladder carcinosarcoma and gallbladder adenocarcinoma (10). CSGB should be suspected when CT imaging shows a large tumor size and calcification within the tumor and the gallbladder morphology remains unchanged (11, 12). Joao Cruz et al. reported that CSGB showed equal to low intensity on T1-weighted images and non-uniform and moderate to high intensity on T2-weighted images, which may be a specific feature to distinguish CSGB from other gallbladder malignancies (13). Whereas PET-CT helps to differentiate between benign and malignant lesions of the gallbladder. The standardized uptake values were higher for malignant lesions (7). Early and accurate detection of CSGB is particularly difficult due to the lack of specific imaging manifestations and serologic markers for CSGB.

Given the lack of specific clinical manifestations and examination findings of CSGB, the rate of preoperative misdiagnosis is high, relying on surgery or sampling histopathology to confirm the diagnosis, It is recognized by the inclusion of both cancerous and sarcomatous components and may present positive for cytokeratin and vimentin (1, 7). The cancerous epithelial component of gallbladder carcinosarcoma is mostly adenocarcinoma, with squamous cell carcinoma also present, and the mesenchymal component ranges from homogeneous sarcoma to more ectopic components such as malignant bone, cartilage, and other mesenchymal tissues (11). Mochizuki et al. reported a case of gallbladder carcinosarcoma composed of adenocarcinoma, neuroendocrine carcinoma, undifferentiated carcinoma, and other specific pathologic elements (4). SMA, EMA, P53 and desmin can also be positive in some patients (1). The appropriate procedure should be selected according to the depth and extent of CSGB tumor infiltration. In general, If the tumor is confined to the submucosal layer, simple cholecystectomy is the better option. If confined to the gallbladder, cholecystectomy and removal of liver tissue around the gallbladder is an option, and complete resection of advanced gallbladder carcinosarcoma remains more difficult (10). However, even with surgical treatment, the prognosis of patients with CSGB is still unsatisfactory, higher risk of local recurrence and metastasis to the liver, lymph nodes, and peritoneum (14). A study conducted by Okabayashi et al. found that the overall 5-year survival rate for patients treated surgically for gallbladder carcinosarcoma was 31.0% and the hospital mortality rate was 8.3%, which was worse than gallbladder adenocarcinoma (11). We analyzed data from 19 patients recorded in the literature in this period from 2000 to 2024 who underwent surgical management for carcinosarcoma of the gall bladder (**Table 1**). These patients consisted of 3 males and 16 females with an age range 40–88 years.

**Table 1:** Nineteen reported cases of surgical resection for carcinosarcoma of the gall bladder from 2000 to 2024.

Authors	Published year	Age (years)	Sex	Initial symptoms	Size (mm)	Stage	Surgical procedure	Perioperative medical treatment	Survival
Kunio Mochizuki (4)	2019	88	F	chill, tremor and vomiting	60 × 25 mm	Not described	cholecystectomy with biliary and abdominal drainages without lymph node dissection and choledocholithotomy	None	10 months
Madani Ayoub (25)	2020	66	M	upper abdominal pain spreading to the scapula and the right shoulder associated with vomiting	170× 110 × 70 mm	pT4N0M0 IVA	extended gallbladder cholecystectomy	None	6 months after a decline of 12 months, no recurrence was detected.
Kevin L (26)	2005	64	F	nausea, vomiting,and right upper quadrant pain	120 × 100 × 70 mm	Not described	A cholecystectomy with wedge resection of the gallbladder fossa (involving liver segments 4 and 5)	None	2 months
Jeffrey J Pu (10)	2011	59	F	worsening right upper quadrant abdominal dull pain	110 × 25 × 22 mm	T1bN0M0	hepatocholangojejunostomy Roux-en-Y	adjuvant chemotherapy (oxaliplatin 150 mg and 5-Fu 500 mg six cycles)	Subsequent 6-month follow-ups were not abnormal
Jumana A. Alratroot (5)	2019	52	F	right upper quadrant pain accompanied by vomiting	110x60mm	pT4N2Mx III	radical cholecystectomy	adjuvant chemotherapy (gemcitabine 1000 mg/m2 and cisplatin 25 mg/m2)	Not described
Toru Kato (27)	2022	71	F	jaundice	50 × 37× 22 mm	T3N2M0 IVB	pancreaticoduodenectomy with gallbladder bed resection of the liver	gemcitabine, cisplatin, and tegafur/gimeracil/oteracil potassium (GCS)	8 months
Pallavi Prasad (6)	2022	44	F	dull and non-radiating abdominal pain	75 ×43 ×35 mm	IIb	laparotomy and extended cholecystectomy with liver wedge resection	Not described	Not described
Menka Khanna (3)	2013	45	F	severe pain in the right hypochondrium	25 x15mm	Not described	cholecystectomy, with a wedge resection of the underlying liver tissue	Not described	Subsequent 6-months follow-ups were not abnormal
Ruhaid Khurram (28)	2020	64	F	abdominal pain, distension, intermittent fevers	215 mm × 135 mm × 145 mm	Not described	surgical resection via a central hepatectomy and a cholecystectomy	Not described	Not described
YAN WANG (29)	2013	68	F	right upper abdominal pain and jaundice	100x70x50mm	Not described	cholecystectomy with liver segmentectomy (S4a+S5) and a lymph node dissection	Not described	Not described
JOÃO CRUZ (13)	2016	52	F	right upper abdominal discomfort and vomiting	Not described	Not described	extended cholecystectomy	None	5 days
Faten Limaiem (30)	2023	78	F	right upper quadrant pain	Not described	pT3N0M0	cholecystectomy, hepatic bisegmentectomy, and lymph node dissection	Not described	Not described
Mohammed Yousef Aldossary (31)	2019	40	M	severe intermittent pain in the right hypochondrium	130mm	pT3N0M1 IVB	radical cholecystectomy with excision of the tumour, and resection of segment V	2 cycles of adjuvant chemotherapy	3 months
		52	F	right upper quadrant pain	110mm	pT4N2M1 IVB	radical cholecystectomy, transverse colectomy, and distal gastrectomy with Roux-en-Y gastrojejunostomy	None	1 month
Sung Bae Park (32)	2011	77	F	severe right upper quadrant abdominal pain	78 × 55 × 12 mm	T2N1M0 IIIB	cholecystectomy, which included resection of 3 to 5 cm wedge of liver tissue at the gallbladder bed	None	2 months
Yi Dai (33)	2024	65	M	right upper abdomen distending pain and discomfort	80 × 70 × 50mm.	GxT2NxM0 IIb	Hepatectomy and cholecystectomy	Not described	1 month
Hiroyuki Matsubayashi (8)	2019	72	F	nausea and abdominal pain	84×72 mm	IIIA	extended cholecystectomy	Not described	Subsequent 73-months follow-ups were not abnormal
Aqdas A. Al Omran (34)	2021	63	F	right hypochondrium abdominal pain	Not described	pT2aN1Mx	extended cholecystectomy with complete porta hepatis lymph node dissection	Not described	Not described
K Kataria (35)	2012	55	F	right upper quadrant pain, anorexia, and weight loss	70x50x30mm	Not described	cholecystectomy & wedge resection of the liver with resection of transverse colon and paraduodenal lymph nodes	None	Not described

F, female; M, male.

Postoperative adjuvant therapy is often recommended for patients with CSGB in order to eliminate or control potential micrometastatic lesions and to slow the progression of the patient’s disease. Chen et al. analyzed 11 patients with CSGB who received adjuvant chemotherapy, with gemcitabine or cisplatin- based chemotherapy protocols being the more common chemotherapy protocols, The patients who received chemotherapy had a 9-month longer median survival than those who did not receive adjuvant chemotherapy, suggesting that adjuvant chemotherapy may improve the prognosis of patients to some extent, but the limitation of this study is that the sample size was small and did not allow for adequate elucidation of the benefits of chemotherapy (1). Targeted therapies such as anlotinib can also be used in patients with CSGB and have shown promising efficacy, which may be a potential treatment in the future. Previous cases of gemcitabine combined with oxaliplatin (GEMOX protocol) with anti-PD-1 therapy for CSGB to achieve complete response have been reported (15). Few cases of radiotherapy for CSGB have been reported and its efficacy is still debatable.

The KRAS gene belongs to the RAS family, which is one of the common oncogenes in cancer, with a variety of mutation sites, the most common of which include G12C, G12D, and G12V. This patient has a relatively rare KRAS G12V mutation, suggestive of a pathogenic mutation, but no corresponding drug is currently on the market. Currently, CSGB patients with the KRAS G12V mutation are relatively rare, Patients with tumors containing the KRAS G12V mutation generally have a poorer prognosis. KRAS mutations in patients with pancreatic cancer tissue are correlated with poorer disease-free survival and OS, and the KRAS G12V mutation (*P* = 0.001) was an independent poor prognostic factor for OS in patients with advanced pancreatic cancer, The KRAS G12V mutation is correlated with high circulating regulatory T cell levels, which are both associated with poorer prognosis in advanced pancreatic cancer patients (16). Evidence from Prajish Iyer et al. suggests that KRAS G12V but not KRAS G13D mutations may prevent gallbladder cancer patients from responding to anti-EGFR therapy, KRAS G13D mutation as sensitive but G12V as resistant to anti-EGFR therapy. Ignoring KRAS-activating mutations may lead to inaccurate patient prognosis (17). Therefore, genetic testing of patients with gallbladder carcinosarcoma is of significance in evaluating the prognosis and drug resistance of patients. The relationship between KRAS G12V mutation and prognosis of patients with gallbladder carcinosarcoma remains to be investigated. Research on inhibitors targeting the KRAS G12C mutation has made remarkable progress, such as Sotorasib, can be used to treat non-small cell lung cancer patients carrying the KRAS G12C mutation (18).There is currently no corresponding drug on the market for the KRAS G12V mutation, but studies are ongoing. For example, a study by Enrica et al. on non-small cell lung cancer with the KRAS G12V mutation reported that the use of VS-6766 and Defactinib, a FAK inhibitor, could show synergistic antitumor activity (19). In addition, the KRAS inhibitor RMC-6236 is similarly effective for a wide range of RAS-mutant tumors including the KRAS G12V mutation, e.g., lung cancer, pancreatic cancer, etc. (20). In the future, it is expected that more inhibitors targeting the KRAS G12V mutation will be researched and developed to provide new therapeutic options for patients carrying such mutations, including CSGB.

Reviewing the postoperative treatment of our patient, taking into account the need to balance both the cancer and sarcoma components in terms of the choice of postoperative chemotherapy protocols for the patient, six cycles of chemotherapy with the more drug-sensitive paclitaxel and isocyclophosphamide were used, and the patient had a disease-free survival of 16 months. A phase III randomized controlled trial randomly assigned 685 patients of unresectable or metastatic BTC to receive either anti-pd-L1 durvalumab or placebo in combination with cisplatin plus gemcitabine for up to 8 cycles, The median overall survival (OS) of patients in the durvalumab and placebo groups was 12.8 and 11.5 months, and the median progression-free survival was 7.2 months (95% CI, 6.7-7.4) and 5.7 months (95% CI, 5.6-6.7), respectively, The rates of grade 3 or 4 adverse events were 75.7% and 77.8%, respectively (21). The patient in this case had a BRCA1 mutation. BRCA1/2 mutations in advanced BTC vary in frequency and type, Some BTC patients with germline BRCA mutations can have better treatment outcomes than BTC patients with somatic mutations, and patients carrying BRCA mutations are more likely to benefit from immunotherapy alone or in combination (22). In pancreatic ductal adenocarcinoma, BRCA1 and BRCA2 proteins are involved in DNA damage recognition and repair by homologous recombination, The mutation results in the inability to repair broken double-stranded DNA and increased sensitivity to cytotoxic drugs such as platinum, and the use of platinum-based chemotherapy protocols improves OS and DFS in patients with BRCA1/BRCA2 mutations (23). Patients with BRCA1 mutations in breast cancer benefit more from platinum-based protocols than patients with BRCA2 mutations, suggesting that patients with BRCA1 mutations may be more sensitive to platinum-based drug therapy (24). Referring to the results of other tumor-related studies and in combination with the patient’s BRCA1 mutation, the patient’s chemotherapy protocol was changed to GP in combination with durvalumab after disease recurrence and metastasis, at the same time, the patient was orally treated with anlotinib, which is effective for sarcoma. Immunotherapy has also been reported to be used in conjunction with chemotherapy in gallbladder carcinosarcoma and achieved good results (15). It is suggested that the application of drugs with different mechanisms of action may serve to improve the efficacy of the combined treatment of the disease and help to improve the state of immunosuppression in the organism, thereby increasing the overall survival rate. Because of the patient’s IV° myelosuppression, chemotherapy was changed to the XELOX protocol containing oxaliplatin, which is more sensitive to the patient, with intermittent oral targeted therapy with anlotinib during the same period. During the treatment period, the patient developed fever, jaundice, and other symptoms of biliary tract infection, and the test results suggested cholestasis, The symptoms of obstruction were well relieved by PTCD, and the complications were actively managed, which improved the quality and survival of patients to a great extent.

## Conclusions

Our patient had a rare gallbladder carcinosarcoma with the KRAS G12V mutation, and available reports suggest that the prognosis for this type of disease is extremely poor. However, through multidisciplinary collaboration and rational application of multiple treatments according to the patient’s own condition, specific site of the tumor, pathological type, and extent of invasion on the basis of evidence-based medicine, the quality of this patient’s survival has been relatively improved and the survival period prolonged. It goes without saying that there are limitations to the reference value of single case report We expect more clinical studies on CSGB to be conducted in the future to standardize the diagnosis and treatment of the disease. It is also anticipated that more studies targeting genetic mutations, including the KRAS G12V mutation, will be conducted in patients with CSGB, providing new treatment options for this patient population.

## Data Availability

The original contributions presented in the study are included in the article/supplementary material. Further inquiries can be directed to the corresponding author.
